# Fast Visual Tracking Based on Convolutional Networks

**DOI:** 10.3390/s18082405

**Published:** 2018-07-24

**Authors:** Ren-Jie Huang, Chun-Yu Tsao, Yi-Pin Kuo, Yi-Chung Lai, Chi Chung Liu, Zhe-Wei Tu, Jung-Hua Wang, Chung-Cheng Chang

**Affiliations:** 1 Department of Electrical Engineering, National Taiwan Ocean University, Keelung City 20224, Taiwan; 20553001@mail.ntou.edu.tw (R.-J.H.); 10453016@mail.ntou.edu.tw (C.-Y.T.); k8130488@gmail.com (Y.-C.L.); free_touch_tim@hotmail.com (C.C.L.); jjay6twtw@gmail.com (Z.-W.T.); ccchang@mail.ntou.edu.tw (C.-C.C.); 2 Ship and Ocean Industries R&D Center (SOIC), New Taipei City 25170, Taiwan; s880062@gmail.com

**Keywords:** visual tracking, convolutional networks, clustering, IoT, object detection

## Abstract

Recently, an upsurge of deep learning has provided a new direction for the field of computer vision and visual tracking. However, expensive offline training time and the large number of images required by deep learning have greatly hindered progress. This paper aims to further improve the computational performance of CNT which is reported to deliver 5 fps performance in visual tracking, we propose a method called Fast-CNT which differs from CNT in three aspects: firstly, an adaptive *k* value (rather than a constant 100) is determined for an input video; secondly, background filters used in CNT are omitted in this work to save computation time without affecting performance; thirdly, SURF feature points are used in conjunction with the particle filter to address the drift problem in CNT. Extensive experimental results on land and undersea video sequences show that Fast-CNT outperforms CNT by 2~10 times in terms of computational efficiency.

## 1. Introduction

The issue of applying deep neural networks to visual tracking has been widely studied both academically and industrially. In general, visual tracking is conducted through comparison of features of a target object and the video image in the search region. The feature extractor plays a key role during the tracking process, and using proper features can dramatically affect the tracking performance. By its nature, feature extraction can be classified into two major categories: feature engineering and feature learning. Feature engineering refers to the process of using domain knowledge to find features inherent to the data useful in the subsequent decision task; normally it is conducted in a hand-crafted manner. Some literature has argued that hand-crafted features may perform better than otherwise in exploiting robust representations for some special cases. However, hand-crafted features suffer a major drawback of being not tailored for all generic objects, and hence require sophisticated learning techniques to improve their representative capabilities. On the other hand, feature learning is characterized by extracting useful image representations through learning. Currently, the most well-known example is the deep learning or deep neural network, a key attribute of which is that features are extracted, along with other interlayer connection weights update, during the training process for the entire network architecture. Many researchers advocate applying deep networks to learn generic representations offline from a huge number of training images, claiming that such generic features possess a better generalization capability and therefore are more effective in distinguishing objects of different categories [1,2]. Despite the fact that deep networks have drawn increased attention with state-of-the-art results in image recognition, classification, detection or even aesthetic evaluation, much less effort has been directed to applying deep networks in visual tracking. This is mainly attributed to insufficient training data, namely, only the target state (i.e., position and size) in the first frame is available for training. Furthermore, visual tracking remains a challenging problem in many practical applications due to factors such as partial occlusion, cluttered background, fast and abrupt motion, dramatic illumination changes, and large variations in viewpoint and pose.

The architecture of deep networks presents a hot research topic, especially convolutional neural networks (CNNs). For example, [1,2] pay special focus on offline learning an effective feature extractor with a large amount of auxiliary data. However, such offline training is time-consuming and the learned generic representation usually is less discriminative for tracking specific objects. To speed up the process, another line of research emphasis on the unsupervised feature learning problem for online visual tracking without training. For instance, the convolutional network-based tracker (CNT) [3] has shown that the similar local structural and inner geometric layout information among the targets over consequent frames are sufficient to achieve 5 fps performance in visual tracking. CNT is characterized by the fact that it requires no offline training with a large amount of auxiliary data, and simple two-layer feed-forward convolutional networks are powerful enough to learn robust image representations for visual tracking. CNT extracts a set of normalized patches from the target region in the first frame and apply the *k*-means algorithm to obtain a fixed bank of target filters. Simultaneously CNT extracts several background samples surrounding the target, and also apply *k*-means to obtain a bank of background filters. Then the fixed target filters are subtracted by the background filters, and subsequently used to convolve each normalized sample extracted from subsequent frames to define a set of feature maps in those subsequent frames. Finally, these feature maps are used to match the target template for finding an optimal candidate during visual tracking. In this way, CNT uses much less training time, compared to the offline learning proposed in [1,2].

The classic *k*-means algorithm in CNT simply sets the *k* value to a constant (100). Both the role of *k*-means clustering in unsupervised feature learning and the effect of varying the value of *k* are not elaborated in [3], yet, it is widely known that an inappropriate *k* value often results in a poor clustering result. In light of this, the proposed Fast-CNT differs from CNT in three main aspects: firstly, an adaptive *k* value (much less than 100) is determined for an input video, doing so can improve computation efficiency. Secondly, unlike in CNT, background filters are abstained in our framework to save computation time without affecting performance. Thirdly SURF [4] feature points are incorporated into the particle filter [5] to address the drift problem in CNT. Results of this work may not only allow us to better understand the significance of *k*-means clustering in unsupervised feature extraction, but also facilitate the assessment of how and to what extent different strategies can be used to improve the original CNT. Last but not least, Fast-CNT can double the computing performance of the original CNT, namely increasing from 5 fps to more than 10 fps.

It has been shown in [3] that the original CNT can stably track the target with much better accuracy than well-known methods like transformation Gaussian progress regression (TGPR) [6], kernel correlation filter (KCF) [7] and context tracker (CXT) [8]. Moreover, in the case when the target object is moving away from the camera, TGPR, KCF, Struck [9] and visual tracker sampler (VTS) [10] cannot perform well as compared to the original CNT. Based on this reason, all the experimental results in this paper will focus on the comparison of Fast-CNT and original CNT.

## 2. Convolutional Network Based Tracker

To understand our work, it is necessary to briefly discuss the original CNT [3]. CNT provides additional useful information for visual tracking without the expensive training time required in conventional convolution networks. In CNT, in order to keep the computational load as low as possible, only one layer of the convolution network is used in the simple layer, where feature operators are obtained from the well-known *k*-means algorithm, namely, each cluster centroid corresponds to a feature operator for the input image. The CNT task can be roughly divided into image representation and the tracking process. 

### 2.1. Image Representation

#### 2.1.1. Preprocessing

First, each input image is rescaled to a canonical size and represented by the corresponding intensity values. Then a bank of overlapping local image patches is sampled pixel by pixel using a sliding window. Each patch is subjected to a subtracting-the-mean operation and L2 normalization that correspond to local brightness and contrast normalization, respectively.

#### 2.1.2. Simple Layer

After preprocessing, the *k*-means algorithm selects a bank of patches sampled from the object region in the first frame as fixed object filters $F^{O}$. The background filters $F^{B}$ are selected by the *k*-means algorithm from background samples surrounding the object in the current frame. Then the fixed object filters are subtracted by the background filters, and the preprocessed object image *I* is convolved with them to obtain the simple feature maps (i.e., simple cells in [3]) defined as:

(1) $$S=\left(F^{O}-F^{B}\right)⊗I\;(⊗=\mathrm{convolution}\;\mathrm{operator})$$

#### 2.1.3. Complex Layer

In order to increase the strength of the simple feature maps, a complex layer is formed by stacking the simple feature maps together to construct a (complex) feature map *C* of a 3D tensor. According to [3], the complex cell features can preserve the geometric layouts of the useful parts at different scales.

#### 2.1.4. Model Update

In order to track a continually moving target, it is necessary to update the target template, CNT utilizes soft shrinkage function to enhance the sparse characteristics of *C*, which makes *C* more robust to appearance variation, and the sparse feature map $\hat{c}$ is defined as:

(2) $$\hat{c}=\mathrm{sign}\left(C\right)\max\left(0,\mathrm{abs}\left(C\right)-\lambda \right)\;\left(\lambda =\mathrm{median}(C)\right)$$

*C* in Equation (2) is updated gradually to adapt to the changes of appearance over time, a temporal low-pass filtering method is adopted to update the target template:

(3) $$c_{t}=\left(1-\rho \right)c_{t-1}+\rho {\hat{c}}_{t-1}$$

where ρ, $c_{t}$, and ${\hat{c}}_{t-1}$ represent the learning parameter, the target template at frame *t*, the sparse feature map of the tracked target at frame *t*.

### 2.2. Tracking Process

The tracking process in CNT is implemented using the particle filtering scheme, and it is based on an optimal Bayesian estimation and Monte Carlo model. More specifically, particles maintain a probability distribution over the state (location, scale, etc.) of the object being tracked. Each particle is a guess representing one possible location of the object being tracked.

For convenience, the object filter set is denoted as $F^{O}$, the background filter set as $F_{t-1}^{B}$, target state set as ${\hat{s}}_{t-1}$, and the target template set as $c_{t}$ when estimating the target state at frame *t*. The CNT tracking algorithm mainly consists of five steps:

1. Sampling *N* candidate particles ${\left\{s_{t}^{i}\right\}}_{i=1}^{N}$.
2. Extracting the preprocessed image patch for each particle $s_{t}^{i}$, subjecting the patch to the simple and complex layer and employing Equations (4) and (5) to obtain the corresponding representation $c_{t}^{i}$, followed by computing similarity between the target template and representation $c_{t}^{i}$.
  (4) $$c_{t}^{i}=C_{t}^{i}☉w\;(☉\;=\;\mathrm{element-wise}\;\mathrm{multiplication})$$
  (5) $$w_{i}=\{\begin{array}{l} 1,\;\;c_{t}\left(i\right)\neq 0\\ 0,\;\;otherwise \end{array}\;$$
3. Estimating the optimal state ${\hat{s}}_{t}$ according to the similarity.
4. Extracting background samples to update the corresponding filters $F_{t}^{B}$ set forth in Equation (1), then computing the sparse representation ${\hat{c}}_{t}$ of the target template using Equations (1) and (2) and using Equation (3) to update the target template $c_{t+1}$.

## 3. Methodology

**Figure 1:**
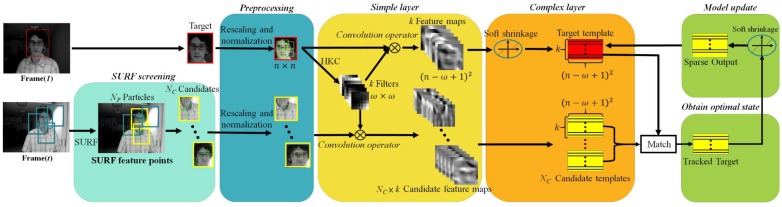
Flowchart of the proposed Fast-CNT.

Figure 1 shows a flowchart of the proposed Fast-CNT. It mainly consists of five steps:

1. Every input frame at arbitrary time *t* (Frame(*t*)), except the first frame, is subjected to the SURF screening. There are *N_P_* particles are sampled from Frame(*t*), each particle corresponds to a blue bounding box with a randomly selected size. The number of SURF feature points (green prints) covered by each blue bounding box (namely, the particle) is checked to determine if the particle in question is qualified as *N_C_* candidates (yellow bounding box).
2. The preprocessing stage rescales and normalizes the target box in Frame(1) as well as the plural candidate boxes in Frame(*t*) into canonical *n* × *n* images, and then in Frame(1) extracts a set of local patches with size of *w* × *w* (i.e., multiple *w* × *w* patches or subimages in the rescaled and normalized target, *w* ≦ *n*). Just like in CNT [3] and in this work, the canonical n is heuristically set to 32, however, both the target box and candidate boxes may have different sizes from each other, thus in the preprocessing stage they are rescaled (corresponding to the “warping” in [3]) to the same size and subjected to L2 normalization. In fact, doing so ensures to achieve the desired effect that one of the *N_C_* sets of feature maps in the simple layer well preserves the local structure of the target and delicately overcome the target appearance changes significantly due to illumination changes and scale variations, as well shown in Figure 2 of [3].
3. In the simple layer, local patches from rescaled and normalized target in Frame(1) are subjected to the HKC (hierarchical *k*-means clustering) algorithm [11] to obtain *k* fixed target filters for convolution(without zero or mirror paddings at the image boundary) with the target to generate *k* feature maps with size of (*n* − *w* + 1)^2^. On the other hand, target filters (*k* filters) are convolved with each rescaled and normalized candidate boxes to generate *N_C_* sets of feature maps, each set has *k* feature maps.
4. In the complex layer, only the *k* target feature maps (not the candidate feature maps) are de-noised by soft shrinkage [12]. Subsequently, *k* simple cell feature maps and *N_C_* × *k* candidate feature maps are stacked to represent the target template and *N_C_* candidate templates.
5. Finally, each *N_C_* candidate template is matched with the target template in order to find optimal candidate state, which is used to update the target template by Equation (3) in the model update block. Note that the matching can be done by, for example, subtracting the target template from each of the *N_C_* candidate templates and selecting one with the minimum result, or through the inner product operation and choosing the one with the greatest similarity, etc.

Compared to CNT, Fast-CNT preserves the characteristic that it requires no offline training with a large amount of auxiliary data, and the major improvements herein are threefold: firstly, unlike the constant *k* (=100) value as set forth in [3], a much smaller *k* value is determined in an automated manner to achieve better clustering result and faster computation. Secondly, we show that background filters used in [3] can be omitted to further enhance the computational performance without sacrificing any tracking accuracy. Thirdly, the incorporation of SURF feature points for screening candidate boxes in the particle filters to improve the drift problems.

### 3.1. Adaptive k Value

The classic *k*-means algorithm is based on Euclidean distance and characterized by (a) the number of clusters *k* needs to be set in advance; (b) input data is assumed to have a globular/convex-like distribution, which means that *k*-means is not applicable to data of non-globular/non-convex clusters with different sizes and shapes. As in [3], *k*-means is applied to obtain feature extractors in this work, we will explore the role of *k*-means in the CNT framework, and how to choose tan appropriate *k* value. Yet, before proceeding, we need to elaborate on the concept of sparse dictionary learning.

Sparse dictionary learning is a representation learning method, which aims at finding dictionaries inferred from input data, and these dictionaries consist of a linear combination of basic elements from input data. The data is mapped to feature subspace through the dictionary, and results of which are encoded by sparse coding in order to find the sparse representation of the input data. In this context, the centroids of clusters obtained from *k*-means can be regarded as dictionary elements which can define features for a supervised learning task [13]. Namely, the dictionary (centroids of clusters) learned by *k*-means serve as a feature extractor in CNT, and it is convolved with the input image to generate feature maps which are similar to features of images learned from a single-layered convolutional neural network, its role is just like a simple cell which is more sensitive to the boundary information with its receptive field in biological vision system. Not only the dictionary is capable of effectively depicting edge information of the input image, but also these feature representations are robust in the sense that they are both scale-invariant and shift-invariant, while maintaining the image local structure.

The number of clusters *k* is heuristically set to be 100 in the original CNT. However, the choice of *k* value should be carefully treated, and an inappropriate *k* value may result in meaningless clustering result. In order to address this problem, numerous studies were surveyed: Agglomerative hierarchical clustering (AHC) [14], DBSCAN [15], HKC [11], and so on. DBSCAN is a density-based clustering algorithm, wherein for each sample point in the data set, the number of sample points *MinPts* within the range of the specified radius *ε* is calculated to estimate the density. DBSCAN is more resistant to noise than *k*-means and can handle clusters of any shape and size. However, it has two hyper parameters (*ε* and *MinPts*) which rely on experiences to control in order to produce good cluster results, making it rather difficult to find a good parameter combination, particularly in the case of high dimensional input data or where the density difference is relatively large. AHC takes each sample as a separate cluster, and then merge the closest pair of clusters until there is only one cluster. The hierarchical clustering has the significant advantage of using any effective distance measurement to achieve clustering, and it can draw its operation into a dendrogram.

HKC is a two-stage algorithm. In the segmenting phase, the data set is divided into multiple clusters through *k*-means. In the merging phase, the single linkage clustering algorithm is used to merge the closest pair of clusters. After *k*-means partitions the input data into several groups, HKC can quickly and effectively produce tree-like clustering results. Therefore, we adopt HKC to find a *k* value that is most suitable for the input images by taking into account the fact that there are various variations in the pixel intensity distribution in the input image, i.e., spatial distribution of the input data itself may have different sizes and densities. Because the classic *k*-means cannot explore clusters of different shapes and sizes, it is not suitable for our purpose here. In contrast, HKC combines the advantages of *k*-means and hierarchical clustering algorithm and overcomes each other’s shortcoming. In the segmenting phase of HKC, the data set is divided into multiple clusters through *k*-means, where the number of clusters is purposely set larger to reduce the effect of noise and outliers on *k*-means. In the merging phase, the single linkage clustering algorithm is used to make up for the shortcoming of *k*-means that cannot explore the clustering of arbitrary shapes, and also provide a readable dendrogram to allow us to study its clustering results.

**Figure 2:**
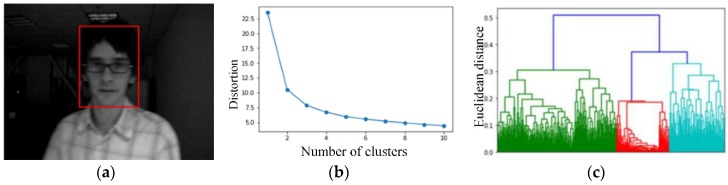
(**a**) Target image; (**b**) Using the Elbow method to check the validity of clusters; (**c**) Three clusters are also determined by HKC, consistent with the result in (**b**).

We note that in order to justify the clustering validity of HKC, the famous Elbow method is used, and Figure 2 shows both the Elbow and HKC determine that the number of clusters is three for the target image in Figure 2a. Therefore, thorough out this work the *k* value is advantageously determined in an automated manner.

### 3.2. SURF Screening

SURF is a scale-invariant method. It is well known that SURF is three times faster than SIFT with comparable performance, and is good at handling images with blurring and rotation, but not good at handling viewpoint changes and illumination changes. In this paper, SURF feature points are used solely for the purpose of screening those bounding boxes qualified for the subsequent tracking task, that is, bounding boxes with sufficient number of SURF feature points will be selected (*N_C_* yellow bounding boxes in Figure 1) and subjected to the subsequent operations of pre-processing, convolution in the simple layer, and matching after the complex layer, etc.

As CNT tracks the target bounding box without any prior knowledge, a great robustness is needed in order to avoid losing the target (i.e., the drift problem). In this work, SURF feature points [4] are first extracted for each frame, then 200 random points generated in the particle filter, each of which corresponds to a bounding box. The number of SURF feature points included in each bounding box is checked if it is greater than a preset threshold. Because unqualified bounding boxes are excluded from being candidate templates, SURF screening can provide a benefit of increasing the accuracy of target tracking.

**Figure 3:**
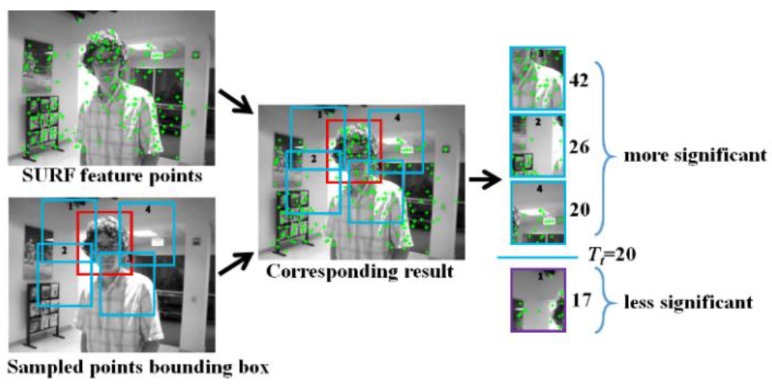
Overview of the SURF-based screening process.

**Figure 4:**
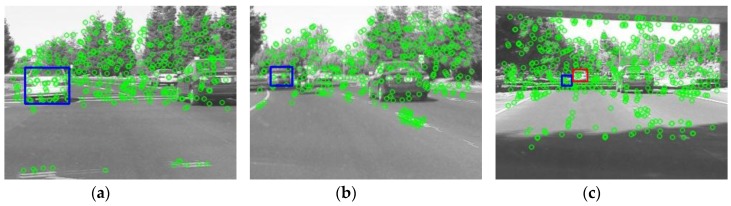
As the size of a target object (white car on the left) shrinks, the target could be erroneously tracked; (**a**) target object; (**b**) the target object is shrinking; (**c**) the red bounding box indicates the erroneous tracking location.

Figure 3 shows how the screening process works, wherein *T_t_* = 20 is a heuristic threshold for checking if a bounding box generated by the particle filter is qualified, i.e., bounding boxes containing less than 20 feature points are discarded. Clearly, using a fixed threshold does not apply to all kinds of input images, as observed in [16] a target object may undergo changeable scale, rotation, perspective, blurs and illumination from frames to frames, causing the number of SURF feature points contained in a specific bounding box varies drastically. Figure 4 shows a situation in which the tracking is prone to miss the target, as the white car on the left (target) is driving away from the viewer, the size of blue bounding boxes in Figure 4a–c shrinks in the video sequences. Note that blue boxes in Figure 4a,b indicate the tracking result is still correct, yet Figure 4c shows that the target car has been erroneously tracked as the red box location, instead of the blue box location. A feasible approach to this problem is to update the threshold dynamically. For example, a bounding box containing less than five feature points, or having the number of pixels constituting the box itself is less than 100 pixels is too small and will be excluded from the subsequent operations. Hence, the threshold *T_t_* is prescribed as:

(6) $$T_{t}=\{\begin{array}{c} \;\;\;\;\;\;\;\;\;\;\;P_{2}/2,\;\;\;\;\;if\;t=\;2,\;3\\ \left(\frac{P_{t-1}}{2}+\frac{P_{t-2}}{2}\right)/2,\;\;\;if\;t>3\;and\;P_{t-1}>5\;and\;{\hat{s}}_{t-1}<100\;pixels\\ \;\;\;\;\;\;\;\;\;\;\;0,\;\;\;\;\;\;\;\;\;\;\;if\;t>3\;and\;P_{t-1}<5\;and\;{\hat{s}}_{t-1}<100\;pixels \end{array}$$

where $T_{t}$ and $N_{t}$ represent the threshold at *t_th_* frame and the number of SURF feature points contained in a target bounding box, respectively.

### 3.3. Abstaining Use of Background Filters

**Figure 5:**
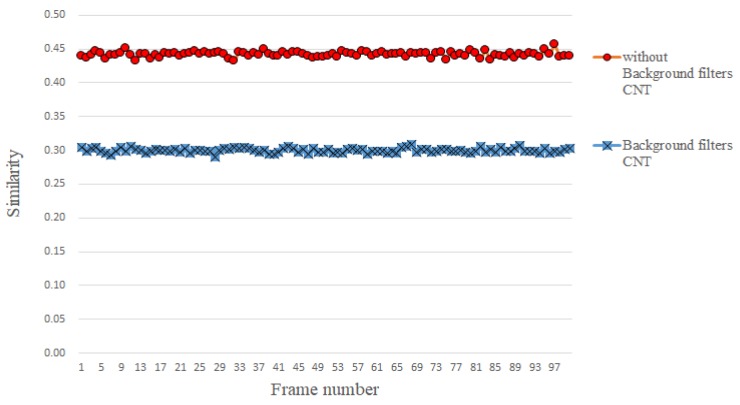
Red line (marked as o) represents the similarity between candidate template and target template when background filters are not used, blue line represents (marked as x) the similarity between candidate template and target template when the background filters are used.

In CNT, background filters are calculated and used for updating the target filters at different frames. However, we conjecture that different filters in the filter bank themselves are capable of extracting various features. When the target filters are subtracted by the background filters in CNT, the extracted features will be inevitably affected, which could incur negative effects on the subsequent template comparison. As shown in Figure 5, in the absence of background filters, the similarity between the candidate template and the target template is actually higher than otherwise. Our experiments show that the tracking accuracy is almost the same, regardless of using background filters or not. This finding equivalently states that using the *k*-means learning the dictionaries (target filter) as a feature extractor is capable of capturing sufficient information for visual tracking. Thus, unlike in [3], this work chooses to abstain from the use of background filters, a direct benefit of doing so is the reduction in the computation time.

## 4. Experimental Results

### 4.1. Experiment Setup

Fast-CNT is implemented in Tensorflow and coded in Python, running on an Intel i7 CPU (2.8 GHz). Computing performance is improved up to 11 fps over the 5 fps in the original CNT. For a fair comparison, the benchmark dataset [17] which includes 50 fully-annotated videos is used, and the experiment setup is mostly the same as the CNT, namely the images of each video are converted to grayscale, and the state of the target in the first frame given by the ground truth, the image rescaled to 32 × 32 (by INTER_AREA-resampling in OpenCV), and the receptive field size set to 6 × 6 (i.e., the filter of *w* × *w* in the Simple layer of Figure 1). For simplicity and without loss of generality, the target state parameters (*σ_x_*, *σ_y_*, *σ_s_*) are assumed independent and modeled by three scalar Gaussian distributions, and hence the particle states updated can be formulated as Brownian motion [18], and the standard deviations of the candidate particle state: *σ_x_* = 4, *σ_y_* = 4, and *σ_s_* = 0.01. However, unlike CNT, the number of filters is decided by HKC, and *N* = 200 particles are used.

### 4.2. Evaluation Metrics

**Figure 6:**
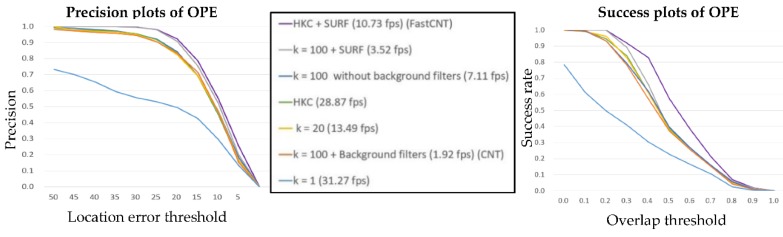
The success and precision plots of OPE for the top 6 experimental trackers.

For quantitative evaluations, the results of one-pass evaluation (OPE) are presented [17], wherein the precision plot shows the percentage of all frames whose estimated location is within the threshold distance of the ground truth, the threshold is set 0~50. Meanwhile, the success plot is based on the bounding box overlap score, given the tracked bounding box *b_t_* and the ground truth bounding box *b_g_*, the overlap score is defined as $S=\frac{\left|b_{t}\cap b_{g}\right|}{\left|b_{t}\cup b_{g}\right|}$. The success plot shows the ratio of successful frames when the threshold is varied from 0 to 1. In order to show the effectiveness of Fast-CNT, we present various experiments using different values of *k* in combination with other constraints. Specifically, seven parametric combinations were tested using the Skater video of the benchmark dataset, each parametric combination representing a combination of a *k* value with or without SURF screening and background filters. The results shown in Figure 6 are obtained by averaging over 10 different runs of each parametric combination.

Three different values (1, 20, and 100) of *k* were tested while setting other parameters at fixed values. The results are shown in Figure 6, and some observations are given below. First, when *k* = 1, as the complex layer contains only a single feature map, resulting in a failure tracking. Second, when *k* = 20 and 100, the performances are much better, indicating the resulting feature representation are sufficient enough. In short, the value of *k* cannot be too small to ensure that the features are sufficiently complex for tracking tasks. On the other hand, the value of *k* needs not to be excessively large, as it will incur higher computational load. In Fast-CNT, HKC is employed to automatically generate a proper *k* value based on input target, the cluster centroids are determined from image patches, and the proper value of *k* is found to be in the range of 3 to 8 for the benchmark dataset used.

Figure 6 clearly shows that the SURF screening can greatly increase the success rate and precision rate of the tracking algorithm, although it will inevitably cost some computational load, and incurs tracking errors in some special circumstances (e.g., shadows). Unlike in CNT, background filters are not used in this work in updating the target filters at different frames. In Figure 6, the success rate and the precision rate of background filters are nearly the same for the first three parametric combinations. However, the computation performance can reach 7.11 fps without using the background filters (3rd combination). In contrast, the original CNT can only deliver 1.92 fps.

### 4.3. Underwater Tracking

Underwater robotics, marine science, and underwater exploration have become more active in recent years. Naturally, there is a strong need to apply computer vision-based algorithms to these works, however, most of the tracking algorithms and data set are used in onshore or land-based applications, and relatively few of them tackles the task of underwater video tracking. Difficulties lie in that many underwater operations require clear and easily-recognizable underwater images, yet given the illumination attenuation, uneven illumination results in lower and unbalanced image brightness. Moreover, there is serious back-scattering noise due to scattering and absorption, and underwater images often suffer from poor quality, such as low contrast, blur, and so on. Thus, it would be interesting to see the performance of Fast-CNT in dealing with undersea video images.

**Figure 7:**
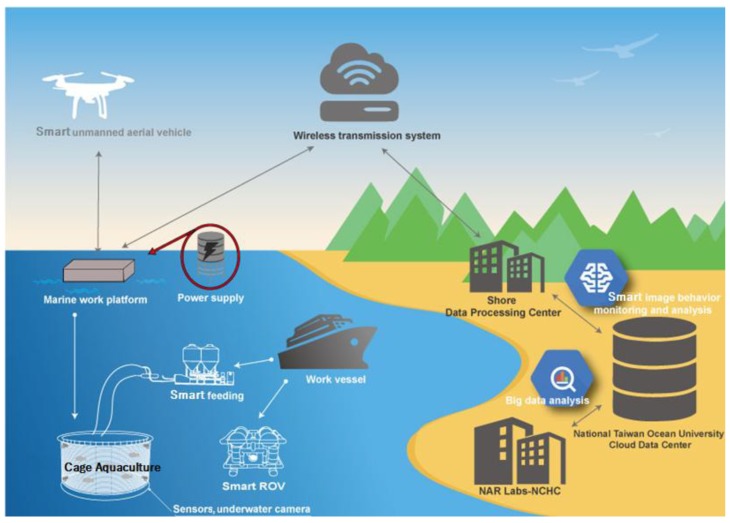
A practical Smart Cage Aquaculture Management System (SCAMS).

As the global population grows, the per capita consumption of aquatic products has been rapidly increasing [19]. In order to meet the market demand for water products, the demand for fishery production in many countries has been largely replaced by fish farming (i.e., aquaculture). To ensure a good harvest, it is important to constantly monitor the health state of farmed fish in the cage. Traditional methods rely on regular manual observations to confirm whether the fish are in the healthy or contagious state, they require a great deal of subjective experience and thus are inefficient and costly. We believe if the fish in the cage can be monitored through IoT technology, e.g., by using various sensors such as underwater camera [20], velocimeter [21], temperature sensor, etc. [22], substantial economic benefits can be obtained by collecting huge amount of long-term breeding and environment data and subjecting them to Big data analysis. Recently, our research team has set forth an AI initiative, which is funded by MOST under the title: *Applying artificial intelligence (AI) techniques to implement a Practical Smart Cage Aquaculture Management System*, the system architecture of which is shown in Figure 7. Bearing this in mind, the tracking algorithm of this work has been applied to the tracking of the fish farmed in the cage for identifying their health status by analyzing their behavior or swimming gestures.

**Figure 8:**
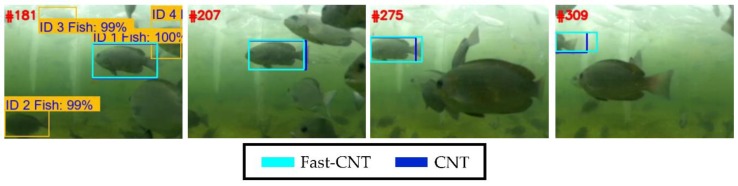
Undersea fish tracking results of Fast-CNT and CNT.

We have tested many underwater videos shot from culture cages, Figure 8 shows an illustrative exemplar tracking result, wherein the target fish is automatically determined using the detection results of the Faster R-CNN [23], which is a deep learning approach effective for object detection. Specifically, a fish detected with the highest confidence is picked as the target fish for tracking, in the case of Figure 8, the fish with 100% confidence and swimming from right to left (i.e., ID1 fish) is picked as the target. We see that although both Fast-CNT and the original CNT can track the target, their averaged computation performances differ quite a lot: 17.9 fps for Fast-CNT and 1.8 fps for the original CNT. Herein, the much better performance of Fast-CNT over the original CNT can be explained as follows: in [3], rather conservative parameters of *N* = 600 particles and *k* value = 100 are used in conjunction with Background Filters, these settings altogether greatly add the computational burdens. In contrast, Fast-CNT uses *N* = 200 particles, *k* value = 8 and abstaining from the use of Background Filters. Note that SURF screening in both trackers are disabled as it is found that very few salient feature points can be extracted for sleek objects such as fish. Experimental parameters settings for the two trackers are shown in Table 1.

## 5. Conclusions

We have successfully improved CNT [3] by modifying both its architecture and implementation, and we found that the *k*-means algorithm in effect serves as an effective dictionary learning scheme for extracting image features in CNT. Furthermore, we have shown that, in terms of architecture, background filters are in effect not necessary in CNT, thus this architectural portion is literally eliminated in Fast-CNT to save computation time without affecting performance. Last but not the least, in order to solve the drift problem, the number of SURF feature points covered by each bounding boxes is checked to determine which bounding boxes are qualified as candidates.

Experimental results using benchmark database video and undersea videos show that Fast-CNT outperforms CNT by 2~10 times in terms of computational efficiency. For future works, because both Fast-CNT and CNT are based on the incremental learning approach of [18], in which the target state parameters are three independent scalar Gaussian distributions and formulated as Brownian motion, and yet no elaborated analysis on how it affects the tracking accuracy as compared to the image representation, we believe this constitutes a worthy research direction.

## Figures and Tables

**Table 1:** Experimental parameters of Fast-CNT and CNT used in Figure 8.

	Fast-CNT	CNT
CPU	Intel i7 7700K 4.20 GHz	
Video resolution	1280 × 960 Downsized to 320 × 240	
Number of Particles	200	600
*k* value	8	100
SURF Screening	Disabled	Disabled
Background Filters	Disabled	Enabled
